# Glucose Metabolic Trapping in Mouse Arteries: Nonradioactive Assay of Atherosclerotic Plaque Inflammation Applicable to Drug Discovery

**DOI:** 10.1371/journal.pone.0050349

**Published:** 2012-11-28

**Authors:** Richard G. Conway, Eyassu Chernet, David C. De Rosa, Robert J. Benschop, Anne B. Need, Emily C. Collins, James S. Bean, J. Michael Kalbfleisch, Mark D. Rekhter

**Affiliations:** 1 Cardiometabolic Diseases and Diabetic Complications, Lilly Research Laboratories, Eli Lilly and Company, Indianapolis, Indiana, United States of America; 2 Psychiatric Disorders, Lilly Research Laboratories, Eli Lilly and Company, Indianapolis, Indiana, United States of America; 3 ImmunoModulation, Lilly Research Laboratories, Eli Lilly and Company, Indianapolis, Indiana, United States of America; 4 Translational Science, Lilly Research Laboratories, Eli Lilly and Company, Indianapolis, Indiana, United States of America; Harvard Medical School, United States of America

## Abstract

**Background:**

^18^F-Fluorodeoxyglucose (FDG)-positron emission tomography (PET) imaging of atherosclerosis in the clinic is based on preferential accumulation of radioactive glucose analog in atherosclerotic plaques. FDG-PET is challenging in mouse models due to limited resolution and high cost. We aimed to quantify accumulation of nonradioactive glucose metabolite, FDG-6-phosphate, in the mouse atherosclerotic plaques as a simple alternative to PET imaging.

**Methodology/Principal Findings:**

Nonradioactive FDG was injected 30 minutes before euthanasia. Arteries were dissected, and lipids were extracted. The arteries were re-extracted with 50% acetonitrile-50% methanol-0.1% formic acid. A daughter ion of FDG-6-phosphate was quantified using liquid chromatography and mass spectrometry (LC/MS/MS). Thus, both traditional (cholesterol) and novel (FDG-6-phosphate) markers were assayed in the same tissue. FDG-6-phosphate was accumulated in atherosclerotic lesions associated with carotid ligation of the Western diet fed ApoE knockout mice (5.9 times increase compare to unligated carotids, p<0.001). Treatment with the liver X receptor agonist T0901317 significantly (2.1 times, p<0.01) reduced FDG-6-phosphate accumulation 2 weeks after surgery. Anti-atherosclerotic effects were independently confirmed by reduction in lesion size, macrophage number, cholesterol ester accumulation, and macrophage proteolytic activity.

**Conclusions/Significance:**

Mass spectrometry of FDG-6-phosphate in experimental atherosclerosis is consistent with plaque inflammation and provides potential translational link to the clinical studies utilizing FDG-PET imaging.

## Introduction

Vascular inflammation plays a critical role in development and rupture of atherosclerotic plaques [1]. Novel anti-inflammatory therapies are actively pursued for treatment of atherosclerosis [2]. That puts visualization and quantification of vascular inflammation to the forefront of drug discovery and development efforts [3]. However, back-and-forth translation of pre-clinical and clinical data remains challenging [1], [4].

FDG-PET imaging is widely used for evaluation of inflammation in human atherosclerotic lesions [5], [6]. FDG accumulation in human atherosclerotic plaques correlates with multiple cardiovascular risk factors and markers of systemic inflammation [7], [8], [9]. PET signal quickly responds to pharmacological treatment, e.g. with the statins [10], [11], [12], thereby making this imaging modality very attractive for clinical testing of experimental drugs.

Ironically, clinical applications of FDG-PET are more reliable than its use in the preclinical space. While it provided valuable and reproducible information in the rabbit models of atherosclerosis [13], [14], [15], [16], FDG-PET application to the most popular mouse models of the disease has been far more challenging [17], [18]. Small size of mouse arteries and close proximity to the heart, that is highly metabolically active, make FDG-PET imaging difficult due to limited resolution. In general, PET imaging is associated with considerable logistic (preparation and handling of a short-lived radioactive isotope, etc.) and financial burden that often makes its application prohibitive for routine drug discovery.

To overcome these problems, we sought an alternative analytical approach. Clinical PET application is based on the hypothesis that plaque macrophages metabolize glucose more actively that other cell types and surrounding tissues [5]. We aimed to (a) develop a technique that would also quantify glucose “metabolic trapping” in atherosclerotic plaques but would not require PET imaging, and (b) validate this technique by demonstrating vascular response to a pharmaceutical with known anti-atherogenic properties.

**Figure 1:**
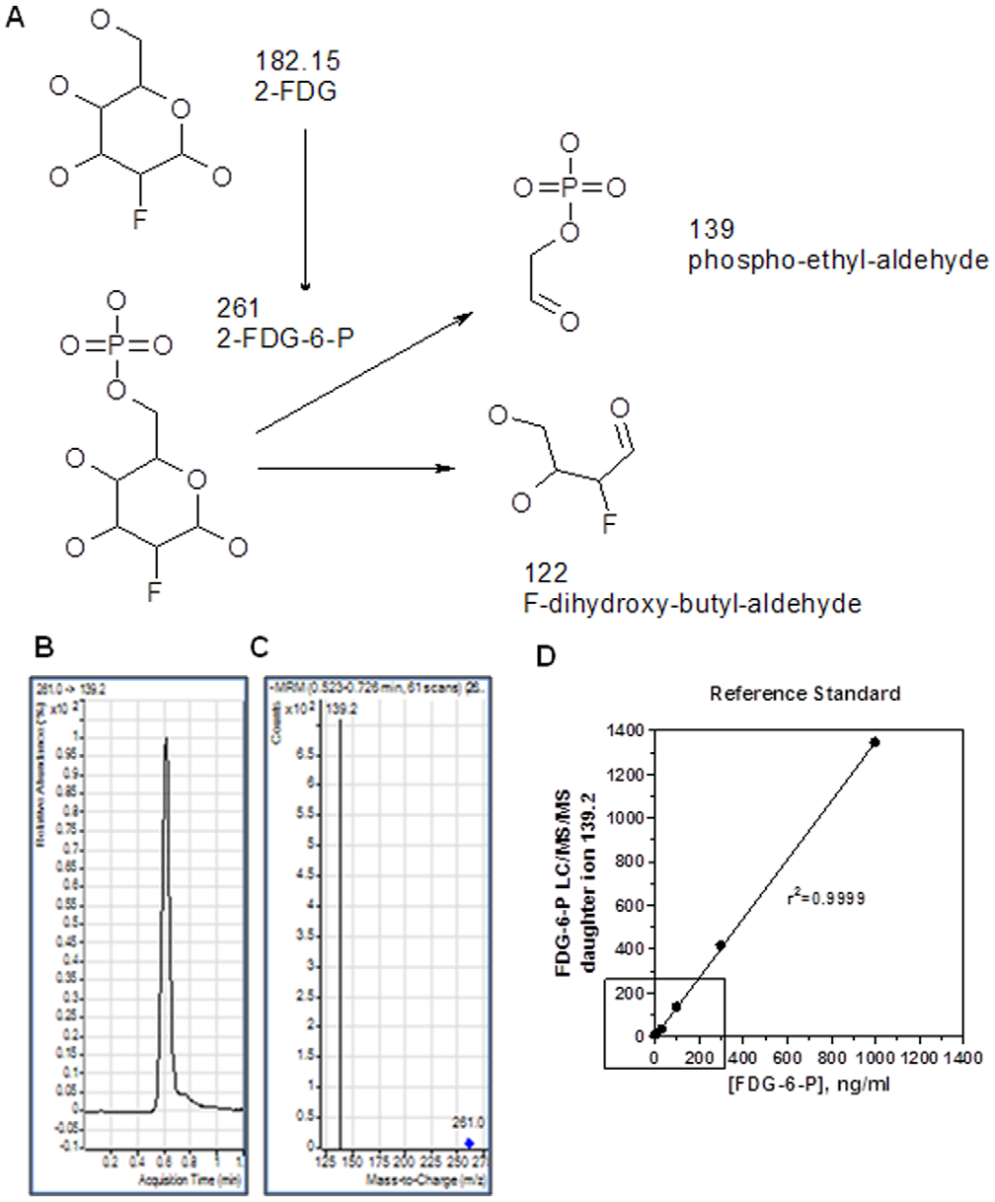
Characterization of FDG-6-P daughter ion. Panel **A** illustrates the working hypothesis, while panels **B**, **C** and **D** represent experimental data. **A**, Predicted FDG-6-P fragmentation pattern. Assuming that FDG metabolism is similar to glucose, it was predicted that FDG-6-P would be generated *in vivo*. In turn, FDG-6-P is likely to generate two products with molecular mass of 139 and 122 respectively, in the process of mass spectrometry. Experimental identification of these products would be indicative of *in vivo* FDG phosphorylation. **B**, Retention time after injection of FDG-6-P standard to the LC/MS/MS **C**, Identification of a peak associated with daughter ion of 139.2 mass-to-charge (m/z) units after injection of FDG-6-P standard to the LC/MS/MS **D**, A standard curve demonstrating proportionality between amount of daughter ion species, 139.2 m/z, and its parent molecule, FDG-6-P.

We have developed a novel quantitative method that is based on evaluation of FDG-6-phosphate (FGG-6-P), a metabolite of FDG, in the mouse arteries. Non-radioactive FDG was injected *in vivo*, and FDG-6-phosphate (FGG-6-P), specifically, a prominent daughter ion of 139.2 (mass to charge ratio), was quantified in arterial extracts using liquid chromatography and mass spectrometry (LC/MS/MS). FDG-6-P levels were substantially increased in atherosclerotic plaques of ApoE KO mice. Treatment with the liver X receptor (LXR) agonist T0901317 reduced FDG-6-P accumulation in concert with reduction of macrophage and cholesterol ester accumulation. We have also demonstrated applicability of FDG-6-P LC/MS/MS for *in vitro* studies.

**Figure 2:**
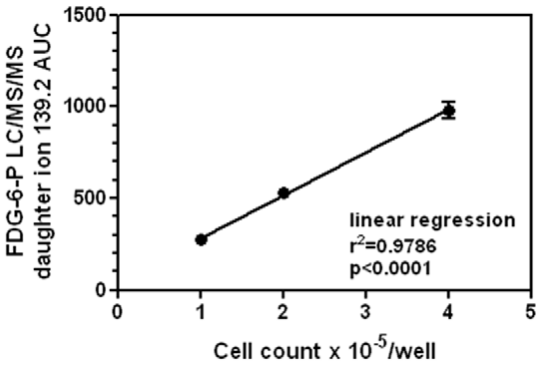
FDG-6-P accumulation in human macrophage cell line (THP-1 cells) *in vitro*. The cells were plated in triplicate in a 96 well plate at different concentrations and incubated with 5.5 mM FDG for 2 hours in glucose-free media. FDG-6-P was measured in cells lysates. FDG-6-P accumulation was proportional to the cell number.

## Methods

### Material

All chemicals used in these studies were reagent grade. FDG (F-5006) and FDG-6-P (F-6037) were from Sigma-Aldrich (St. Louis, MO, USA). RPMI 1640 was from Hyclone (Logan, UT, USA) and 10% FBS was from Invitrogen (Carlsbad, CA, USA). ProSense750 was from VisEn (Woburn, MA, USA).

**Figure 3:**
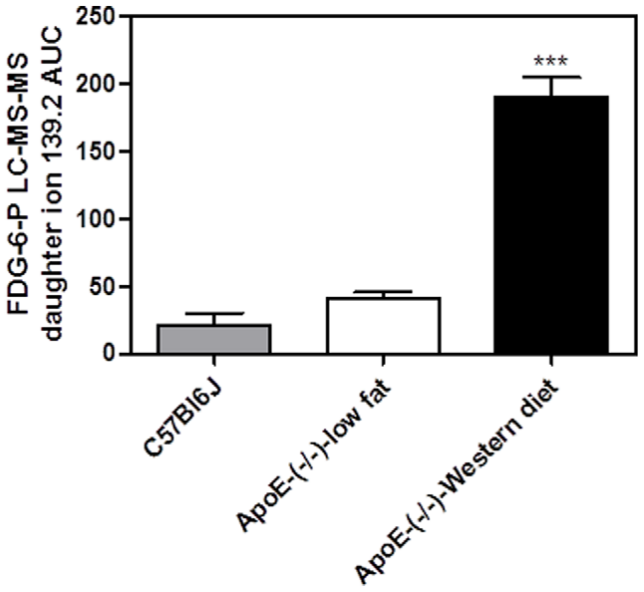
FDG-6-P accumulation in aortas of ApoE KO mice with spontaneous atherosclerosis. FDG was injected intravenously 30 min before euthanasia at the dose of 30 mg/kg to the age-matched normal control (C57BL6) mice that were fed normal chow, ApoE KO mice fed normal chow and Western diet-fed ApoE KO mice (3 months on the diet). Western diet group was significantly different from other groups (one way ANOVA p<0.001; Tukey's test *** p<0.001; n = 6, 6, 5 respectively).

### Cell culture

THP-1 cells were obtained from ATCC. The cells were cultured in RPMI 1640+10% FBS +1% anti-anti (Invitrogen) at 37°C, 5% CO_2_. Cells were harvested non-enzymatically with enzyme free cell dissociation solution (Specialty Media) and resuspended in RPMI 1640 without glucose. Cell concentration was determined using hemocytometer with trypan blue exclusion. Cells were plated in triplicate in a 96 well plate at different concentrations (1, 0.5, or 0.25×106 cells/ml) in 200 µl RPMI +10% FBS +5.5 mM FDG and incubated for 2 hours at 37°C, 5% CO_2_. After incubation, the media was removed, and the cells were lysed in 150 µl MeOH/CH_3_CN/formic acid. The organic lysates were stored covered at 4°C until assayed for FDG-6-P by mass spectrometry. An identical set of wells was prepared and used to measure protein concentration; at the end of the incubation cells were lysed in 50 µl radioimmunoprecipitation assay buffer and protein concentration determined using a BCA protein assay kit (ThermoScientific). Protein concentration was used to normalize FDG-6-P data.

**Figure 4:**
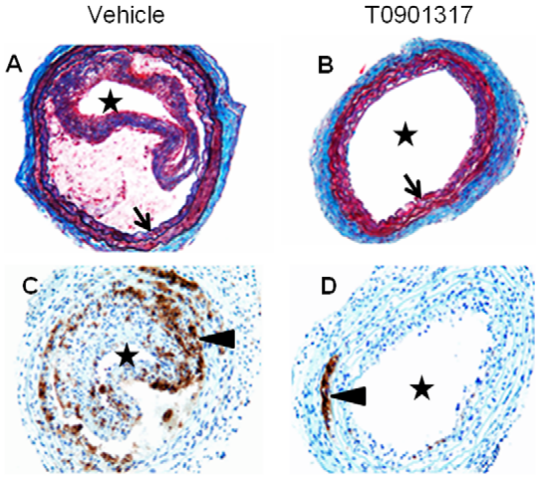
Effects of T0901317on macrophage accumulation in ligated carotid arteries of Western diet fed ApoE KO mice. To accelerate lesion formation, ApoE KO mice were pre-fed with the Western diet for 14 days, and then their left common carotid arteries were ligated. Mice were kept on the same diet following the ligation surgery and were euthanized 14 days after ligation. One group of mice was treated with an LXR agonist T0901317 (10 mg/kg, once daily by oral gavage). A compound was administered for two weeks, starting at the day of ligation. **A, B,** Masson trichrome-elastin staining, 10x objective. Asterisk indicates arterial lumen, arrow indicates internal elastic lamina. Compound treatment dramatically reduced plaque formation. **C, D,** Mac-2 immunostaining, Hemotoxylin nuclear counterstaining, 20x objective. Macrophages exhibit brown cytoplasmic staining (shown by arrowheads). Asterisk indicates arterial lumen. **A** and **C**, vehicle treatment; **B** and **D**, T0901317 treatment. Note drastic reduction of macrophage accumulation in the compound-treated group.

**Figure 5:**
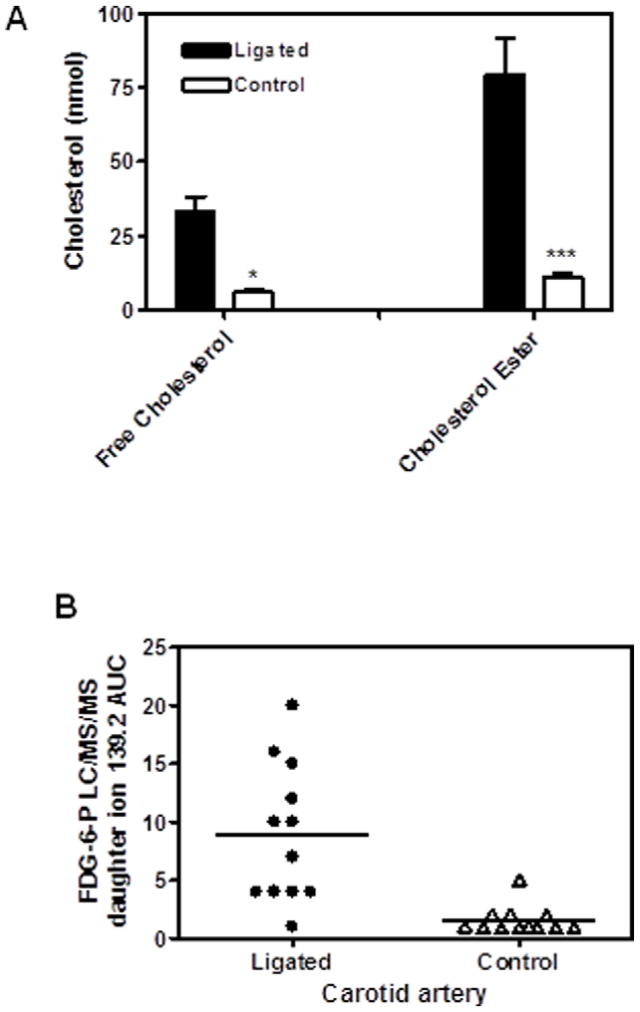
Accumulation of cholesterol (A) and FDG-6-P (B) in ligated and contralateral carotid arteries of ApoE KO mice. ApoE KO mice were pre-fed with the Western diet for 14 days, and then their left common carotid arteries were ligated. Mice were kept on the same diet following the ligation surgery and were euthanized 14 days after ligation. FDG solution was administered at the dose of 30 mg/kg via tail vein injection 30 min before euthanasia. Carotid arteries were dissected and sequentially extracted. Cholesterol and FDG-6-P were measured in the extracts derived from the same vessels. All analytes were compared between ligated and non-ligated (contralatereal) carotid arteries within the same animals using pared t-test. Free cholesterol, cholesterol esters and DFG-6-P were preferentially accumulated in the ligated arteries. Free cholesterol *p<0.05 (n = 11); Cholesterol ester *** p<0.001 (n = 11); FDG-6-P 2-tailed paired t-test p = 0.0011 (n = 12).

### Animal models

The investigation conforms to the *Guide for the Care and Use of Laboratory Animals* published by the US National Institutes of Health (NIH Publication No. 85-23, revised 1996). Experimental procedures using animals were approved by the Eli Lilly Institutional Animal Care and Use Committee. ApoE KO and C57Bl6J mice were from Taconic (Hudson, NY, USA).

**Figure 6:**
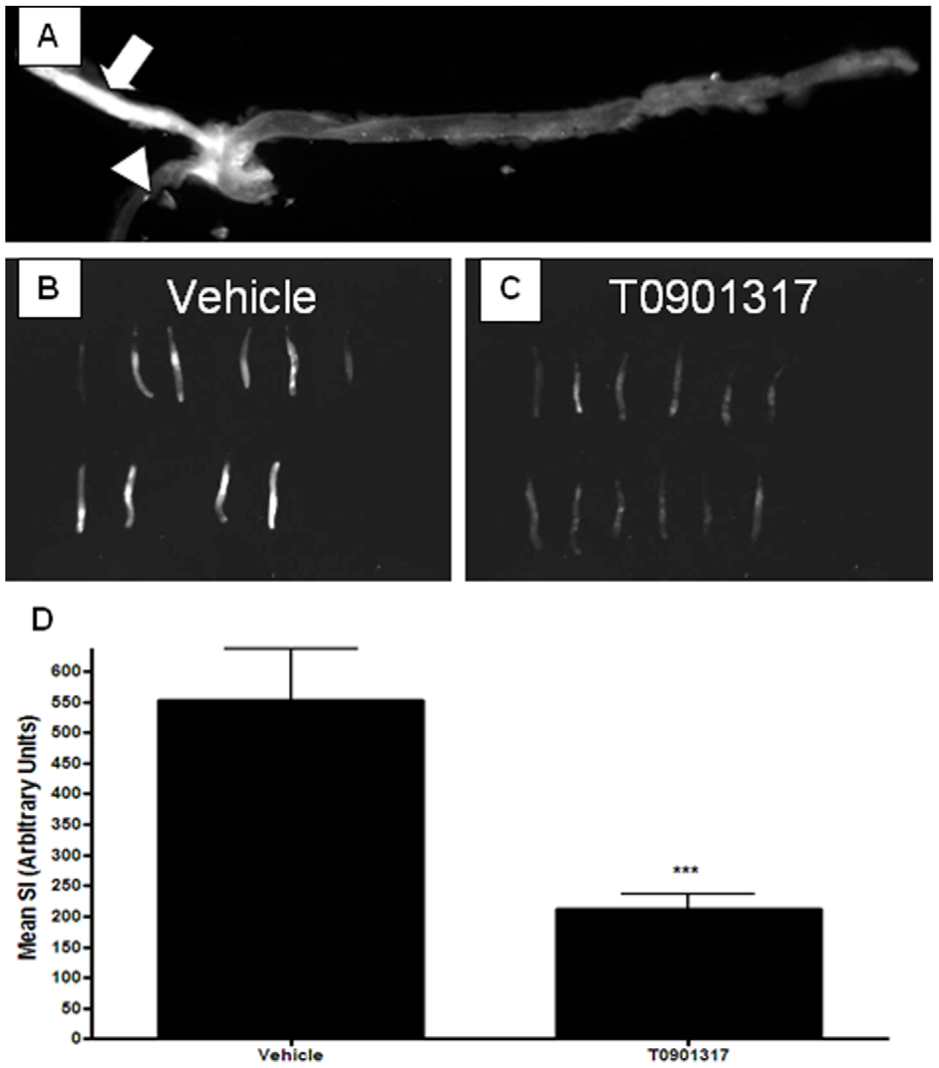
NIRF signal in ligated carotid arteries of Western diet fed ApoE KO mice was reduced after LXR agonist T0901317 treatment. ApoE KO mice were fed Western diet for 2 weeks, and left common carotid arteries were ligated. Animals were maintained on the same diet for another 2 weeks until euthanasia. One group of mice was treated with an LXR agonist T0901317 (10 mg/kg, once daily by oral gavage). A compound was administered for two weeks, starting at the day of ligation. ProSense750was injected intravenously 24 hr before euthanasia, and imaging was performed *ex vivo*. **A**, Note the strong NIRF signal in the ligated (left) common carotid artery (shown by arrow) and lack of fluorescence in the nonligated (right) carotid (shown by arrowhead). Images of ProSense750 NIRF signal in isolated ligated carotid arteries from the animals treated with **B**, vehicle (n = 10) and **C**, T0901317 (n = 12). **D**, signal intensity measurements of the images shown in panels **B** and **C**. T0901317 treatment significantly reduced protease activity in ligated arteries (*** p<0.001).

To evaluate FDG-6-P in spontaneous atherosclerotic lesions, one group of ApoE KO mice (n = 6) was fed standard chow for 3 months, while another group (n = 5) was fed Western diet containing 0.21% cholesterol and 21% fat for the same period of time. Age-matched C57BL6/J mice (n = 6) were fed normal chow and served as a control.

**Figure 7:**
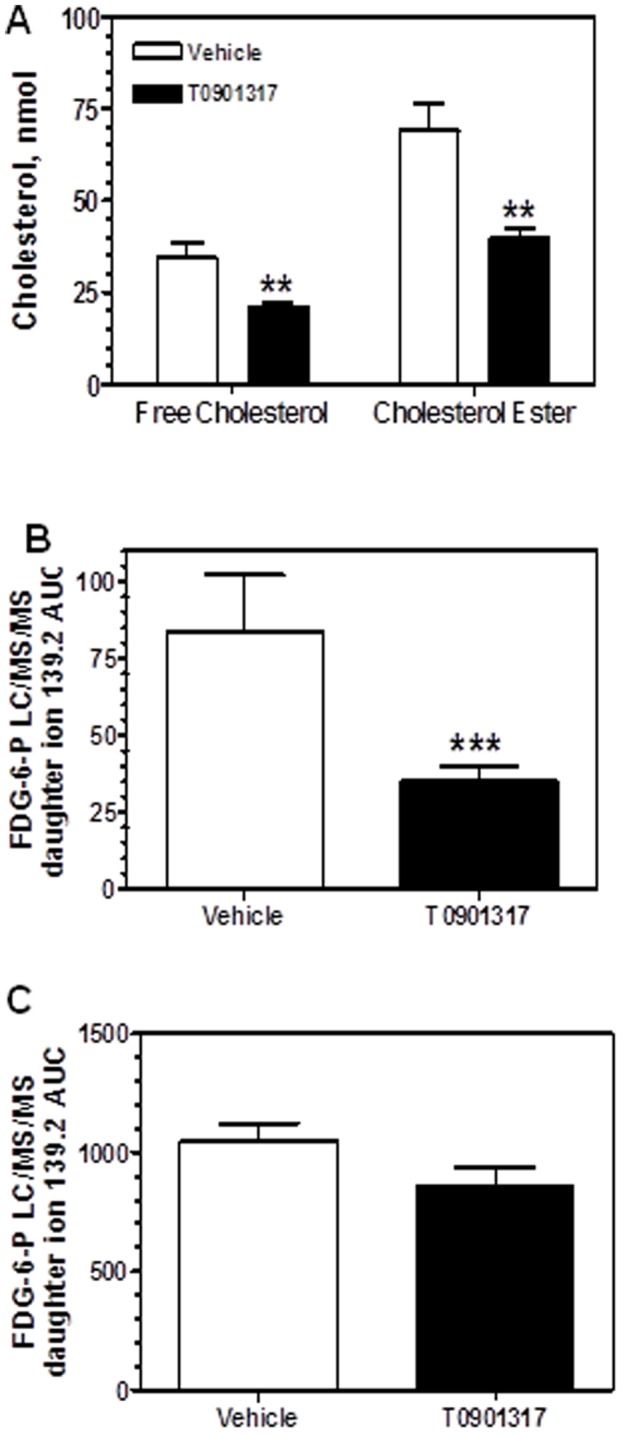
Effects of LXR agonist T0901317 on cholesterol and FDG accumulation in ligated carotid arteries of ApoE KO mice. ApoE KO mice were fed Western diet for 2 weeks, and left common carotid arteries were ligated. Animals were maintained on the same diet for another 2 weeks until euthanasia. One group of mice was treated with an LXR agonist T0901317 (10 mg/kg, once daily by oral gavage). A compound was administered for two weeks, starting at the day of ligation. T0901317 treatment reduced accumulation of cholesterol (**A**) and FDG-6-P (**B**) in ligated carotid arteries of ApoE KO mice. All analytes were measured in the same tissue samples. Free cholesterol and cholesterol esters, p<0.01, n = 12. FDG-6-P, p<0.001; n = 11 **C,** accumulation of FDG-6-P did not change in the spleens from the same animals, indicating the lack of generalized, non-specific effects on resident macrophages.

To accelerate lesion formation, 7-week old ApoE KO mice were pre-fed with the Western diet for 14 days, and then their left common carotid arteries were ligated under isofluorane anesthesia as described previously [19]. Mice were kept on the same diet following the ligation surgery and were euthanized 14 days after ligation by carbon dioxide asphyxiation. 10–12 animals per group were utilized for various experimental protocols, including the drug treatment.

### Drug treatment

To optimize the dose of the compound, T0901317 was administered daily at doses of 0, 3, 10, or 30 mg/kg of weight by oral gavage (10 ml/kg). Vehicle used was 1% hydroxyethylcellulose and 0.25% Tween80 and was similarly administered to control animals. A compound was administered for two weeks, starting at the day of ligation. After obtaining the dose response data, all follow-up experiments have been done with the 10 mg/kg dose of T0901317. This dose was used to evaluate various analytical endpoints described below.

### Near-infrared fluorescence (NIRF) imaging

To obtain an independent measurement of macrophage activation in accelerated atherosclerotic lesions, the carotid arteries from ApoE KO mice on Western diet with carotid ligation were imaged *ex vivo* with the imaging probe ProSense750. ProSense750is activated by proteases, including Cathepsin B, L and S, and transitions from a non-fluorescent to fluorescent form upon activation. Compound treatment experiments were conducted as described above. 24 hours before euthanasia, ProSense750 (2 nmol in 150 µL of PBS) was administered via the tail vein. On the day of imaging, the animals were perfused with saline via the left ventricle. Ligated and unligated (contra-lateral) common carotid arteries were dissected, cleaned and imaged. NIRF reflectance was measured using the Maestro multi-spectral instrument (CRi (now PerkinElmer), Hopkinton, MA) deep red filter set (750 nm lp) with a 3000 ms exposure and 2×2 binning. Data was analyzed using the Maestro Software 2.4.2 and ImageJ.

### Tissue cholesterol analysis

Dissected carotid arteries were immersed in 2∶1 chloroform/methanol, and lipids were extracted overnight. Free cholesterol and cholesterol esters were analyzed by LC/MS/MS as described previously [19]. Delipidated tissues were either embedded into paraffin for further histological analysis or re-extracted for FDG-6-P analysis (vide infra).

### Histology

For histology, 10 equally spaced (200 µm) paraffin cross sections were stained using modified Masson's trichrome procedure that included elastin staining. Macrophages were visualized immunohistochemically using MAC-2 (DAKO). Trichrome-stained sections spanning the entire length of the artery (3 per artery) were used for morphometric analysis. Lesion area was calculated using Image-Pro Plus Version 5.0.1. The lesion area was defined as the region between the lumen and the internal elastic lamina.

### FDG administration

FDG was dissolved in saline to a final concentration of 10 mg/ml. FDG solution was administered at the dose of 30 mg/kg via tail vein injection 30 min before euthanasia. Selection of dose and timing is based on the preliminary experiments (data not shown). After the FDG injection, animals had access to food and water ad libitum. As described above, the mice were euthanized by carbon dioxide asphyxiation. The animals were perfused with saline via the left ventricle. After perfusion, aortas from the mice with spontaneous atherosclerosis as well as ligated and unligated (contralateral) common carotid arteries from the mice with accelerated atherosclerosis were dissected. The arteries were placed in chloroform:methanol (2∶1) overnight for lipid extraction. Other tissues (e.g. spleen) were frozen on dry ice.

### FDG-6-P extraction

Preliminary studies were conducted by spiking authentic FDG-6-P into biological matrices followed by extraction with various solvents. The method entails extraction of tissue samples with 4X (by weight) volumes of 50% acetonitrile-50% methanol-0.1% formic acid. Tissues were extracted with sonication. Homogenate was centrifuged and clear supernatants transferred to vials or microtiter plate for LC/MS/MS analysis (using 5–10 µl of extract). In the case of mouse carotid artery (dry weight <10 µg) we used 150 µl of extraction solvent to prepare analytical samples.

### FDG-6-P mass spectrometry

The analysis of 2-FDG-6-P was carried out using an Agilent 6140 series triple quad LC/MS/MS with MassHunter data analysis software (Agilent Technologies, Inc., Santa Clara, CA, USA) fitted with an electrospray ion source and run in negative ion mode. Detection was accomplished by monitoring the precursor ion of 2-FDG-6-P with mass-to-charge ratio (m/z) of 261 and targeting its product ion with m/z set to 139.2. The chromatographic separation employed a Cogent Diamond Hydride HPLC column, 4 µm, 100 A, 100 mm×2.1 mm ID (from MicroSolv Technology Corp., Eatontown, NJ, USA) and a mobile phase consisting of 3% acetonitrile in water with an overall 0.1% formic acid content with a flow rate of 0.75 ml/minute. Clearly delineated chromatographic peaks with the retention time of authentic standards and expected molecular mass were seen after each injection of sample. Analytes were quantified based on the area of these peaks.

### Statistics

All values are expressed as mean ± SEM. Dose response and time course data were analyzed using one-way ANOVA. Comparisons of the left (ligated) and right (unaffected) carotid arteries in a mouse model of accelerated atherosclerosis were made by a two-tailed, paired t-test, because pairs of plaque-covered and plaque-free arteries were always obtained from the same animal. Comparisons of ligated arteries from different animals were assessed using an unpaired t-test.

## Results

### LC/MS/MS based quantification of FDG-6-P standard

Since glucose is metabolized by hexokinase into glucose-6-phosphate, we assumed that FDG is metabolized into FDG-6-P via the same route. We also predicted that, in the process of mass spectrometry, FDG-6-P is fragmented into phospho-ethyl-aldehyde with molecular mass of 139 and F-dihydroxy-butyl-aldehyde with molecular mass of 122 (Figure 1A). Experimentally, injection of FDG-6-P standard to the LC/MS/MS resulted in the detection of a strong signal of a daughter ion of 139.2 mass to charge (m/z) units with a retention time of about 0.45 minutes (Figures 1B and 1C). Thus, this daughter ion was identified as a specific product of FDG-6-P. A standard curve (Figure 1D) demonstrated that amount of the daughter ion species was proportional to the amount of its parent molecule, FDG-6-P.

### FDG-6-P accumulation in cell culture

FDG-6-P readily accumulated in the macrophage cell line (THP-1 cells) *in vitro* when FDG was added to the culture media (Figure 2). FDG-6-P accumulation was proportional to the cell number (r = 0.98). Thus, non-radioactive FDG can be a valuable tool for cell culture studies. *In vitro* data also suggested that analogous measurements in the animal tissues are likely to correlate in a linear manner with the number of macrophages in atherosclerotic plaques.

### FDG-6-P accumulation in the arteries of ApoE KO mice with spontaneous and accelerated atherosclerosis

As a result of optimization experiments (data not shown), we selected the regimen when FDG was injected intravenously 30 min before euthanasia at the dose of 30 mg/kg. In a pilot study, to determine accumulation of FDG-6-P in the spontaneous atherosclerotic plaques, FDG was dosed to the age-matched normal control (C57BL6) mice that were fed normal chow, ApoE KO mice fed normal chow and Western diet-fed ApoE KO mice. Development of atherosclerosis was associated with significant increase of FDG-6-P amount in the extracts of normal chow-fed ApoE KO mice and dramatic accumulation in aortas of ApoE KO mice with the diet-induced increase in the plaque coverage (Figure 3).

Detailed analysis of FDG-6-P accumulation was performed in the carotid arteries of Western diet fed ApoE KO mice where atherosclerosis was accelerated by ligation of the left common carotid artery. Briefly, ApoE KO mice were fed Western diet for 2 weeks, and then left common carotid artery underwent ligation. The animals were kept on the Western diet and euthanized in 2 weeks after carotid ligation. Two weeks after flow cessation, lesions (Figure 4A) developed only in the ligated, but not in the contralateral carotid artery. That was reflected by accumulation of cholesterol ester and free cholesterol (Figure 5A) as well as Mac-2 positive macrophages (Figure 4C) in the ligated carotids. Accordingly, FDG-6-P accumulation was dramatically increased in the ligated carotid arteries (Figure 5B).

In order to test if FDG-6-P accumulation in accelerated atherosclerotic lesions correlated with the presence of activated macrophages, we opted to detect the latter by a totally independent technique. A separate set of mice was injected with a NIRF probe Prosense. This probe produces fluorescence when it is proteolytically modified by the macrophage enzymes [20]. Figure 6A demonstrates that Prosense signal was detected only in ligated but not in the contralateral carotids. Thus, FDG-6-P accumulation was associated with accumulation of activated macrophages, free cholesterol and cholesterol esters in ligated carotid arteries of high fat-fed ApoE-KO mice.

### FDG-6-P in accelerated atherosclerotic lesions: Response to experimental therapy

To evaluate if FDG-6-P could be used in experimental pharmacology as a marker of anti-atherosclerotic drug effects, we focused on response of accelerated atherosclerotic lesions to the LXR agonist, T0901317. This compound consistently demonstrated anti-atherogenic activity in spontaneous mouse lesions [21]. First, we tested if those effects were reproducible in the model of accelerated atherosclerosis. A compound was administered for two weeks, starting at the day of ligation. Cholesterol ester accumulation in the lesions was used as the most established characteristic of atherosclerotic plaque. Dose-dependent reduction of cholesterol ester accumulation in carotid lesions 2 weeks after ligation was demonstrated. Cholesterol ester content was reduced from 92.7±18.2 nM in a vehicle group to 63.95±11.4 nm, 37.8±6.8 nM, and 30.6±6 nM in the groups treated with compound at the doses of 3 mg/kg, 10 mg/kg and 30 mg/kg, respectively (all significantly different from the vehicle group). Based on that data, a dose of 10 mg/kg/day was selected for in-depth analysis of compound effects on FDG-6-P accumulation and other relevant analytical endpoints.

The treatment led to dramatic reduction in lesion thickness (Figure 4A and B) that decreased from 42408± 3519 µm2 to 20404±2507 µm2 (p<0.01). Macrophage number in the intima, as measured immunohistochemically (Figure 4C and D), was reduced from 44±8 per section in the vehicle group to 1.6±0.9 in the compound-treated group (p<0.01). Macrophage proteolytic activity was also significantly reduced (Figure 6B, C, D). As mentioned above, cholesterol ester accumulation was significantly reduced (Figure 7A). Finally, and most importantly, these changes were associated with significant decrease in FDG-6-P accumulation in carotid lesions (Figure 7B). Compound treatment did not have any effect on FDG-6-P in the other macrophage-rich tissues in the same animals, e.g. spleen (Figure 7C) thereby demonstrating specific response of atherosclerotic lesions. Thus, FDG-6-P reduction was consistent with the overall anti-atherogenic effects of T0901317.

## Discussion

### Methodological considerations

FDG-PET imaging is widely used for evaluation of vascular inflammation in human atherosclerosis [5], [6]. PET signal quickly responds to pharmacological treatment, e.g. with the statins [10], [11], [12], that makes this imaging modality very attractive for clinical testing of experimental drugs. However, ironically, clinical applications of FDG-PET are more reliable than its use in the preclinical space, especially in the mouse models of atherosclerosis. Small size of mouse arteries and close proximity to the heart, that is highly metabolically active, make FDG-PET imaging problematic due to limited sensitivity and spatial resolution.

To overcome these problems, we sought an alternative analytical strategy. Our novel approach is connected to the clinical modality by similar molecular nature of the quantitative endpoint rather than by similarity of technological platforms. Since clinical PET application is based on the hypothesis that plaque macrophages metabolize glucose more actively that other cell types and surrounding tissues [5], we decided to develop a technique that would also quantify glucose metabolism in atherosclerotic plaques but would not require PET imaging.

We have developed a novel quantitative method that is based on evaluation of FDG-6-P accumulation in the arteries. Non-radioactive FDG is injected *in vivo*, and FDG-6-P, specifically, a prominent daughter ion of 139.2 (m/z) is quantified in the extracts of isolated arteries using LC/MS/MS. Present approach is fundamentally different from previous studies that used positron emitter [18F] FDG and PET detection. The critical difference is that the mass spectrometer is tuned to detect exclusively 139.2 daughter ion of FDG-6-P. It does not detect FDG-6-P itself or the very abundant precursor FDG. As a result, there is virtually no background in the LC/MS/MS experiments.

Importantly, FDG-6-P was successfully extracted from delipidated arteries. We have recently developed a method of cholesterol measurement in atherosclerotic plaques followed by histology of the same sample [19]. The first step of that technique consists of lipid extraction that is subsequently used for cholesterol analysis by LC/MS/MS while delipidated arteries are processed for histology. In the current study, we used the same first step, but delipidated arteries were utilized for extraction and analysis of FDG-6-P. The latter did not interfere with evaluation of atherosclerosis in the same artery by more traditional means, e.g. cholesterol ester accumulation. Sequential extraction of different analytes from the same tissue improves accuracy and allows reducing the number of experimental animals that is associated with obvious humane, logistic and financial benefits.

### Preclinical pharmacology application

We have demonstrated that FDG-6-P accumulation was significantly increased in aortas of Western diet-fed ApoE KO mice (a model of spontaneous atherosclerosis) as well as in ligated carotid arteries of ApoE KO mice (a model of accelerated atherosclerosis). The signal was associated with accumulation of macrophages and cholesterol esters. That observation is consistent with the correlations found in human and rabbit atherosclerotic lesions [15], [16], [22], [23], [24]. Moreover, FDG-6-P accumulation was in concert with an independent measure of macrophage activation, namely their proteolytic activity. Such co-localization is in agreement with the gene expression pattern described in human carotid plaques. 18-FDG uptake was associated with up-regulation of genes encoding proteolytic enzymes, e.g. MMP9 and cathepsin K [25].

Although FDG-PET imaging has been previously performed in ApoE KO mice, these data were controversial. Laurberg et al [17] reported that FDG imaging signal in the aortic arch area primarily originated in the interscapular brown fat, and that the presence of atherosclerotic lesions failed to increase the signal. Other studies that used *ex vivo* autoradiography or scintillation counting demonstrated, however, increase in FDG uptake in aortas of ApoE-KO mice with atherosclerosis [26], [27], [28]. Technical reasons for these discrepancies have been thoroughly discussed [18]. From our perspective, though, FDG-PET imaging approach to quantification of mouse atherosclerosis might be inherently challenging due to its limited spatial resolution and high background levels produced by the heart tissue with very active glucose metabolism. LC/MS/MS measurement of FDG-6-P accumulation can provide considerably better level of precision that is especially critical for evaluation of experimental drug effects.

To explore if FDG-6-P accumulation was sensitive to pharmacological treatment, we used an LXR agonist T0901317 with previously established anti-atherogenic properties [21]. Indeed, T0901317 significantly reduced FDG-6-P accumulation in the ligated carotid arteries. FDG signal reduction occurred in concert with decrease in conventional readouts of atherosclerosis, e.g. cholesterol ester deposition, lesion area and number of macrophages quantified on Mac-2 immunostained slides. T0901317 also significantly decreased NIRF signal associated with accumulation and activation of Prosense, a probe that reflects the presence of “activated” macrophages as defined by their proteinase (primarily cathepsin B) activity [20].

Interestingly, both independent functional methods demonstrated about 50% of signal reduction in the compound-treated group. However, quantification of macrophages immunostained with Mac-2 antibody revealed far more dramatic decrease in macrophage accumulation. This quantitative discrepancy suggests that other cell types associated with mouse atherosclerotic plaques are likely to both actively metabolize glucose and possess proteolytic activity. Alternatively, recently published data suggest that various factors of the microenvironment, notably hypoxia, might regulate macrophage glucose metabolism [29], [30]. It is likely that complex interplay between plaque inflammation as manifested by macrophage number and phenotype and local tissue conditions, e.g. hypoxia and neovascularizarion, as well as some yet unknown variables contribute to FDG signal in the plaque. Therefore, more conservative interpretation of the data is that FDG-6-P accumulation correlated with the number of plaque macrophages but was not exclusively macrophage-specific and/or reflected the changes of the microenvironment that modified glucose metabolism in the plaque.

### Limitations versus benefits

An obvious limitation of our approach is the destructive nature of the assay, which precludes its use *in vivo*. However, from our perspective, this is unlikely to impede its broad appeal to the drug discovery community. In the drug discovery space, a usefulness of a novel pre-clinical assay is primarily evaluated by its ability to increase throughput, improve precision, reduce the number of experiments, reduce an overall cost and deliver better translatability to the clinical measurements.

The main value of *in vivo* imaging is that it facilitates multiple, dynamic evaluations of the same animal/region of interest overtime. Any terminal analysis by definition is lacking this benefit. However, according to our experience, imaging of atherosclerosis in small animals is innately challenging due to the small size of atherosclerotic lesions and their proximity to the heart. *In vivo* imaging approach suffers from the relatively low throughput (a few animals per day) and semi-quantitative output. This is usually sufficient for characterization of the marginal phenotypes in genetically modified mice yet is suboptimal for establishing tight dose response in testing experimental pharmaceuticals. Mass spectrometry of FDG-6-P, on the other hand, is consistent with exquisitely quantitative and reproducible measurements. Moreover, analysis of lipids and FDG-6-P from the same sample allows fast and comprehensive evaluation of anti-atherosclerotic drug effects. We also want to emphasize the pragmatic value of LC/MS/MS in comparison with PET imaging. It is associated with significant cost benefits. Even regardless the price, the vast majority of laboratories, especially affiliated with small biotech companies, contract research organizations and universities do not have instant access to the imaging facilities and synchrotrons necessary for 18FDG production. LC/MS/MS is a routine technique that can be easily applied to evaluation of vascular inflammation.

Traditionally, in the terminal experiments, plaque inflammation is evaluated by quantification of immunostained macrophages. The latter, however, has to be done on the serial sections and thus is extremely labor intensive and time consuming given the number of experimental groups/doses of the compound that are routinely used in the drug discovery studies. LC/MS/MS provides fast (one day) and high throughput analytical alternative. Also, it is likely that glucose trapping is a more generic marker of plaque inflammation that correlates with but is not limited to macrophage accumulation as discussed above. Hence, FDG-6-P accumulation in mouse plaques could be a better predictor of human FDG-PET outcomes than histology.

Another limitation of the proposed methodology is based on the higher amount of nonradioactive FDG injected in mice, whereas much less amount of 18F-FDG is typically used in microPET or PET studies. The pharmacokinetics could be quantitatively different and can affect the sensitivity and specificity of this technique. In the future, it should be compared with 18F-FDG in the same animal model for cross-validation before it can be considered truly translational. However, in its present state, the proposed technique may be used as a first step to plan a drug efficacy study in patients. If an agent fails to reduce *ex vivo* FDG-6-P signal in mice, it is less likely to reduce 18FDG signal in patients.

Taken together, LC/MS/MS based analysis of FDG-6-P accumulation in experimental atherosclerosis can provide a convenient drug discovery tool and useful translational link to the clinical studies utilizing FDG-PET imaging.
